# FGF21-dependent alleviation of cholestasis-induced liver fibrosis by sodium butyrate

**DOI:** 10.3389/fphar.2024.1422770

**Published:** 2024-07-08

**Authors:** Jing Yang, Lei Chen, Shan-Shan Zhao, Chuang Du, Yi-Zhe Fan, Hui-Xin Liu, Yongchun Li, Yong-Zhi Li

**Affiliations:** ^1^ Institute of Life Sciences, China Medical University, Shenyang, China; ^2^ The Sixth Affiliated Hospital of South China University of Technology, Foshan, Guangdong, China; ^3^ Department of Urology, The Fourth Affiliated Hospital of China Medical University, Shenyang, Liaoning, China; ^4^ Liaoning Key Laboratory of Bladder Disease Gene Research, Institute of Health Science, China Medical University, Shenyang, China

**Keywords:** FGF21, cholestasis, liver fibrosis, sodium butyrate, gut microbiota

## Abstract

**Background:**

The beneficial effects of fibroblast growth factor 21 (FGF21) and sodium butyrate (NaB) on protection against cholestasis-induced liver fibrosis are not well known. This study aimed to explore the effects of FGF21 and NaB on bile duct ligation (BDL)-induced liver fibrosis.

**Methods:**

Wild-type (WT) and FGF21 knockout (KO) mice received BDL surgery for 14 days. Liver fibrosis was assessed by Masson’s staining for fibrosis marker expressions at the mRNA or protein levels. Adenovirus-mediated FGF21 overexpression in the WT mice was assessed against BDL damage. BDL surgeries were performed in WT and FGF21 KO mice that were administered either phosphate-buffered saline or NaB. The effects of NaB on the energy metabolism and gut microbiota were assessed using stable metabolism detection and 16S rRNA gene sequencing.

**Results:**

BDL-induced liver fibrosis in the WT mice was accompanied by high induction of FGF21. Compared to the WT mice, the FGF21 KO mice showed more severe liver fibrosis induced by BDL. FGF21 overexpression protected against BDL-induced liver fibrosis, as proved by the decreasing α-SMA at both the mRNA and protein levels. NaB administration enhanced the glucose and energy metabolisms as well as remodeled the gut microbiota. NaB alleviated BDL-induced liver fibrosis in the WT mice but aggravated the same in FGF21 KO mice.

**Conclusion:**

FGF21 plays a key role in alleviating cholestasis-induced liver damage and fibrosis. NaB has beneficial effects on cholestasis in an FGF21-dependent manner. NaB administration can thus be a novel nutritional therapy for treating cholestasis via boosting FGF21 signaling and regulating the gut microbiota.

## Introduction

Cholestasis is a condition characterized by obstruction of bile flow within or outside the liver owing to progressive pathological states, which lead to chronic cholestatic liver diseases; this prevents the bile acids (BAs) from flowing into the small intestine and instead flow into the blood reversely (Penz-Osterreicher et al., 2011; Abshagen et al., 2020). The accumulated BAs in the liver and systemic circulation may then cause damage to the liver and other organs, thereby inducing complications such as liver injury, renal failure, osteoporosis/osteomalacia, portal hypertension, and cholangiocarcinoma that require close follow-up and specific interventions (Jansen et al., 2017). Furthermore, the excess hepatic BAs may cause liver fibrosis and progress to cirrhosis, which in turn could increase mortality as well as affect the quality of life and impose economic burden due to comorbidities associated with liver failure (Liu et al., 2020). Although significant progress has been made in understanding the pathogenesis of cholestasis, the specific mechanisms remain unclear and therapeutic drugs are rare.

Fibroblast growth factor 21 (FGF21) is a stress-induced hormone that exerts beneficial effects in adaptive responses to diverse physiological or pathological stressors, such as starvation, nutrient excess, and mitochondrial stress (Liu et al., 2015; Fisher and Maratos-Flier, 2016; Geng et al., 2020). FGF21 is primarily expressed in the liver and plays an important role in obesity as well as related metabolic diseases. The systemic deficiency of FGF21 has been shown to aggravate liver steatosis and fibrosis in methionine and choline deficient L-amino acid diet (MCD)-fed mice (Fisher et al., 2014), and FGF21 analogues could ameliorate liver injury and fibrosis through multiple mechanisms (Meng et al., 2021; Puengel et al., 2022). In particular, another study revealed that FGF21 can be regulated by the farnesoid X receptor (FXR), which is a well-known nuclear receptor maintaining the homeostasis of BAs, and that the FXR ligand obeticholic acid (OCA) can alleviate liver steatosis as well as fibrosis induced by a western diet by boosting the liver FGF21 signals (Hu et al., 2020). Although these data indicate that FGF21 could act as a therapeutic target in the treatment of liver failure, the beneficial effects of FGF21 have not been assessed under cholestasis conditions (Hu et al., 2020).

Recently, nutritional management was suggested as a therapeutic strategy for cholestatic liver disease (Kloosterman et al., 2022). Nutritional therapy has been demonstrated to play critical roles in multiple chronic liver diseases (Du et al., 2022; Shah and Barritt, 2022; Zhang et al., 2023). Sodium butyrate (NaB) is a well-known short-chain fatty acid (SCFA) with antioxidant capacity and has gained increasing attention for its vast beneficial effects, including modulation of hepatic antioxidation and anti-inflammation (Sun et al., 2018; Tayyeb et al., 2020). Previous works, including ours, indicate that the beneficial effects of NaB on boosting FGF21 signal could be a potential strategy in the treatment of cholestasis-induced liver damage and fibrosis (Li et al., 2012; Erickson and Moreau, 2016; Sheng et al., 2017). In addition, a recent study indicated that NaB administration could regulate the metabolism of liver BAs by modulating the gut microbiota (Ye et al., 2021). However, it remains unknown whether NaB could alleviate cholestasis-induced liver injury and fibrosis; further, it should be identified whether FGF21 is involved in the beneficial effects of NaB.

The present study aimed to examine the beneficial effects of FGF21 and NaB in cholestatic liver disease. We demonstrate that FGF21 could ameliorate cholestasis-induced liver injury and fibrosis and that NaB administration could prevent the progression of bile duct ligation (BDL)-induced liver fibrosis by boosting FGF21 signals and enhancing the host energy metabolism along with modulating the gut microbiota. We also show that the beneficial effects of NaB on cholestasis are dependent on FGF21.

## Materials and methods

### Animal study

All animal protocols in this work were approved by the Institutional Animal Care and Use Committee of China Medical University (CMUXN2022088). Eight-week old male C57BL/6 wild-type (WT) and FGF21 knockout (KO) mice were housed in a specific pathogen-free environment at 20–22°C with 12-h light–dark cycles along with free access to food and water.

BDL is a well-documented technique for inducing cholestasis in animals and was adopted in this study along with procedures described previously (Tag et al., 2015). In brief, the common bile duct was exposed and ligated twice under isoflurane inhalation (1.5 vol%) anesthesia; further, the BDL was not performed for the sham operation mice. For the FGF21 overexpression experiments, the mice were administered Ad-NC (empty control) and Ad-FGF21 (FGF21-overexpressing adenovirus) via tail-vein injection at a dose of 1 × 10^9^ PFU per mouse 3 days before the BDL surgery. The mice were also administered NaB (500 mg/kg bodyweight) daily by oral gavage for 4 weeks before BDL and maintained on NaB administration till euthanization after BDL. The mice were euthanized 14 days after BDL, and their serum, liver, bile, and cecum were collected and stored at −80°C for further analyses.

### Energy metabolism measurement

For the metabolic studies, the mice were housed individually in metabolic cages (Promethion, Sable Systems) and monitored for 24 h after NaB administration for 6 weeks while being allowed free access to food and water. Their food consumption, oxygen consumption, carbon dioxide production, energy expenditure, and respiratory exchange ratio were analyzed by CalR, as described previously (Mina et al., 2018).

### Glucose and insulin tolerance analyses

Glucose tolerance test (GTT) and insulin tolerance test (ITT) were performed as described previously (Geng et al., 2022). Briefly, mice that were fasted overnight were intraperitoneally injected with D-glucose (2 g/kg bodyweight), and the ITT was conducted by injecting insulin (1 U/kg bodyweight) after 6 h of fasting. The blood glucose levels were measured at various time points, as specified in each experiment, using a blood glucose meter.

### Biochemical analysis

The serum alanine aminotransferase (ALT), aspartate transaminase (AST), total bilirubin (TBIL), and total bile acid (TBA) amounts were quantified separately according to manufacturer instructions (Nanjing Jiancheng, China).

### Histological and microscopy analyses

The liver tissues were first fixed in 4% paraformaldehyde and were then embedded in paraffin before being cut into 4-μm-thick slices. Hematoxylin and eosin (H&E) and Masson’s trichrome staining were performed according to standard protocols (Chang et al., 2022). The images of the stained tissues were acquired with a Leica microscope under magnification.

### Gene expression analysis

Liver RNA was isolated using TRIzol (Invitrogen) and reverse transcribed to cDNA (TaKaRa). Real-time quantitative polymerase chain reaction (RT-qPCR) was then performed on a BIO-RAD real-time PCR system using SYBR Green PCR Master Premix (TaKaRa). The mRNA levels were then normalized against the levels of *Gapdh* expressions.

### Western blot analysis

The liver tissues were homogenized on ice in RIPA buffer containing protease inhibitors, phosphatase inhibitors, and EDTA. After centrifugation, the proteins were quantified using a BCA protein assay kit. Equal amounts of the proteins were separated using SDS-PAGE gels and transferred onto polyvinylidene fluoride membranes. After blocking with 5% non-fat milk, the membranes were incubated overnight with primary antibodies at 4°C. The primary antibodies used were TGF-β (ab92486, Abcam), α-SMA (23,779,489, Millipore) Cytokeratin19 (60187-1-lg, Proteintech), and secondary horseradish-peroxidase-conjugated goat anti-rabbit or anti-mouse IgG antibodies (Zhongshan Golden Bridge Biotechnology, China) that were incubated at room temperature for 1 h. After washing in Tris-Buffered Saline and Tween 20 (TBST), the enhanced chemiluminescence (ECL) reagent (Advansta, USA) and Tanon 5200 (Shanghai Tianneng Technology, China) were used to analyze the bands.

### Fecal microbiota 16S rRNA gene sequencing and analysis

The total genome DNA of the cecum content was extracted using hexadecyltrimethylammonium bromide (CTAB), as described previously (Zhao et al., 2022). The V4 hypervariable regions of the bacterial 16S rRNA genes were amplified using specific primers 515F (GTGCCAGCMGCCGCGGTAA) and 806R (GGACTACHVGGGTWTCTAAT). These libraries were sequenced on an Illumina HiSeq platform (Novogene, China) and 250-bp paired-end reads were generated using FLASH (version 1.2.7). The sequencing data were analyzed with QIIME V.2.0. pipeline (Hall and Beiko, 2018), and the Silva 16S rRNA gene reference database was used to classify the Amplicon Sequence Variant (ASV) taxonomically (Quast et al., 2013; Liu et al., 2016). The alpha and beta diversities were analyzed using the R package Vegan, as reported previously (Wang et al., 2018; Liu et al., 2022). Moreover, the predicted functions of the intestinal microbiota were analyzed using PICRUST2 (Douglas et al., 2020).

### Statistical analysis

The data were expressed as means ± standard errors of mean (SEMs). Statistical analyses were carried out using two-tailed unpaired Student’s t-tests for two-group comparisons or one-way analysis of variance (ANOVA) for three or more group comparisons using GraphPad Prism; *p*-values below 0.05 were considered to be statistically significant.

## Results

### FGF21 expression was altered in cholestasis-induced liver fibrosis

**FIGURE 1:**
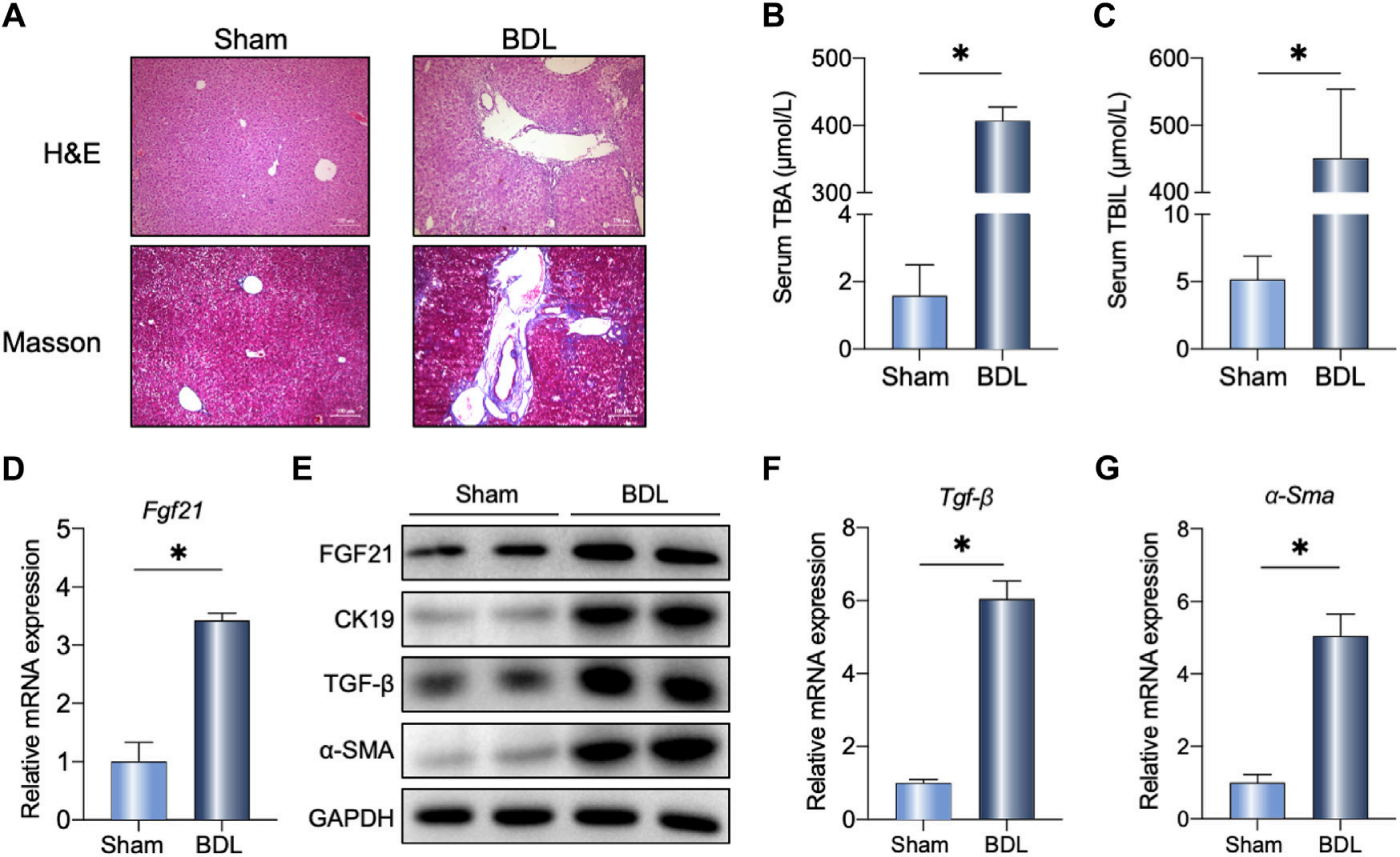
Liver FGF21 induced by bile duct ligation (BDL). **(A)** Representative images of H&E and Masson staining in wild-type (WT) mice 14 days after sham operation or BDL. **(B, C)** Serum total bile acids (TBAs) and total bilirubin (TBIL) in the indicated groups (n = 5). **(D–F)** Relative mRNA expression levels of *Fgf21*, *Tgf-β*, and *α-Sma* in the liver of mice 14 days after either sham operation or BDL. **(G)** Liver protein levels in the two groups. The data were statistically analyzed using unpaired t-test *p < 0.05.

The BDL surgery induced mild liver fibrosis, as shown in Masson’s staining (Figure 1A), accompanied by increased serum TBA and TBIL levels in the mice (Figure 1B, C). As expected, BDL surgery highly induced FGF21 at the mRNA and protein levels (Figure 1D, E). Additionally, we analyzed the single-cell sequencing data of primary sclerosing cholangitis (PSC) patients (shown in Supplementary Figure S1A) and found significant changes in FGF21 expression, indicating the potential of FGF21 as a therapeutic target in the treatment of cholestasis. Compared to the sham operations, the liver fibrosis markers (*Tgf-β* and *α-Sma*) were severely changed at the mRNA levels (Figure 1D–F) after BDL at 14 days. The protein levels of TGF-β, α-SMA, and bile duct hyperplasia marker CK19 were all evaluated in the surgical group and compared with the sham group (Figure 1G).

### Loss of FGF21 aggravated BDL-induced liver fibrosis

**FIGURE 2:**
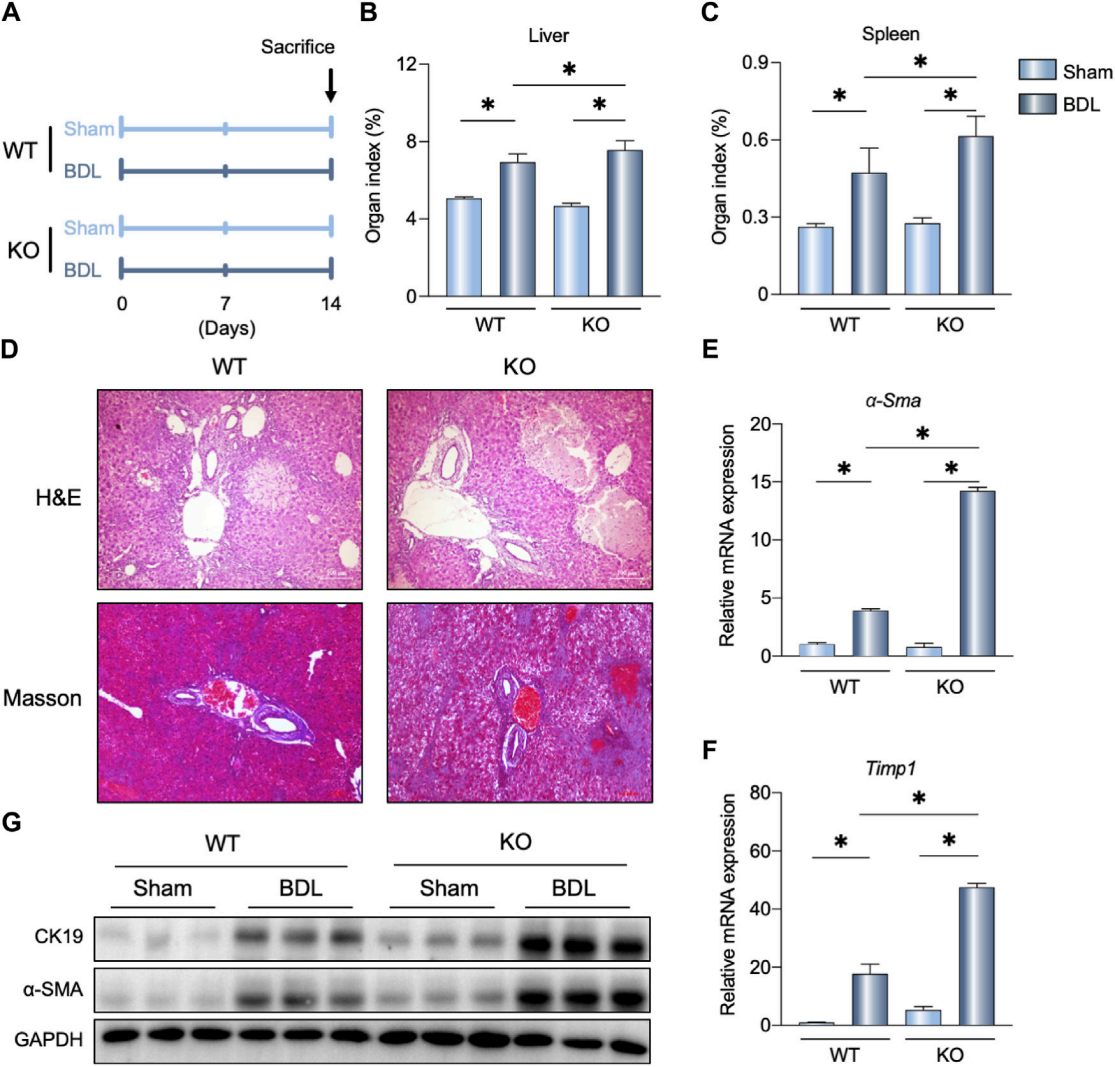
FGF21 knockout (KO) aggravates BDL-induced liver fibrosis. **(A)** Experimental outline (n = 5). **(B, C)** Liver and spleen organ indexes in the indicated groups. **(D)** Representative images of H&E and Masson staining in the WT and FGF21 KO mice 14 days after sham operation or BDL. **(E, F)** Relative mRNA expression levels of *α-Sma* and *Timp1* in the liver of WT and FGF21 KO mice 14 days after either sham operation or BDL. **(G)** Liver protein levels in the four groups. The data were statistically analyzed using ANOVA and Tukey’s multiple comparisons test *p < 0.05.

To determine the physiological role of FGF21 in cholestasis, FGF21 KO mice were subjected to BDL surgery. As shown in Figure 2B, C, increased liver and spleen organ indexes were observed in the FGF21 KO mice after BDL (KO-BDL) compared to the WT mice with BDL (WT-BDL). Histological analyses of the livers showed more necrosis and fibrosis in the KO-BDL mice (Figure 2D). Further, the mRNA levels of *α-Sma* and *Timp1* were increased in the KO-BDL mice (Figure 2E, F); compared to the WT-BDL mice, the protein levels of CK19 and α-SMA were also consistently higher in the KO-BDL mice (Figure 2G).

### FGF21 overexpression improved BDL-induced liver fibrosis

**FIGURE 3:**
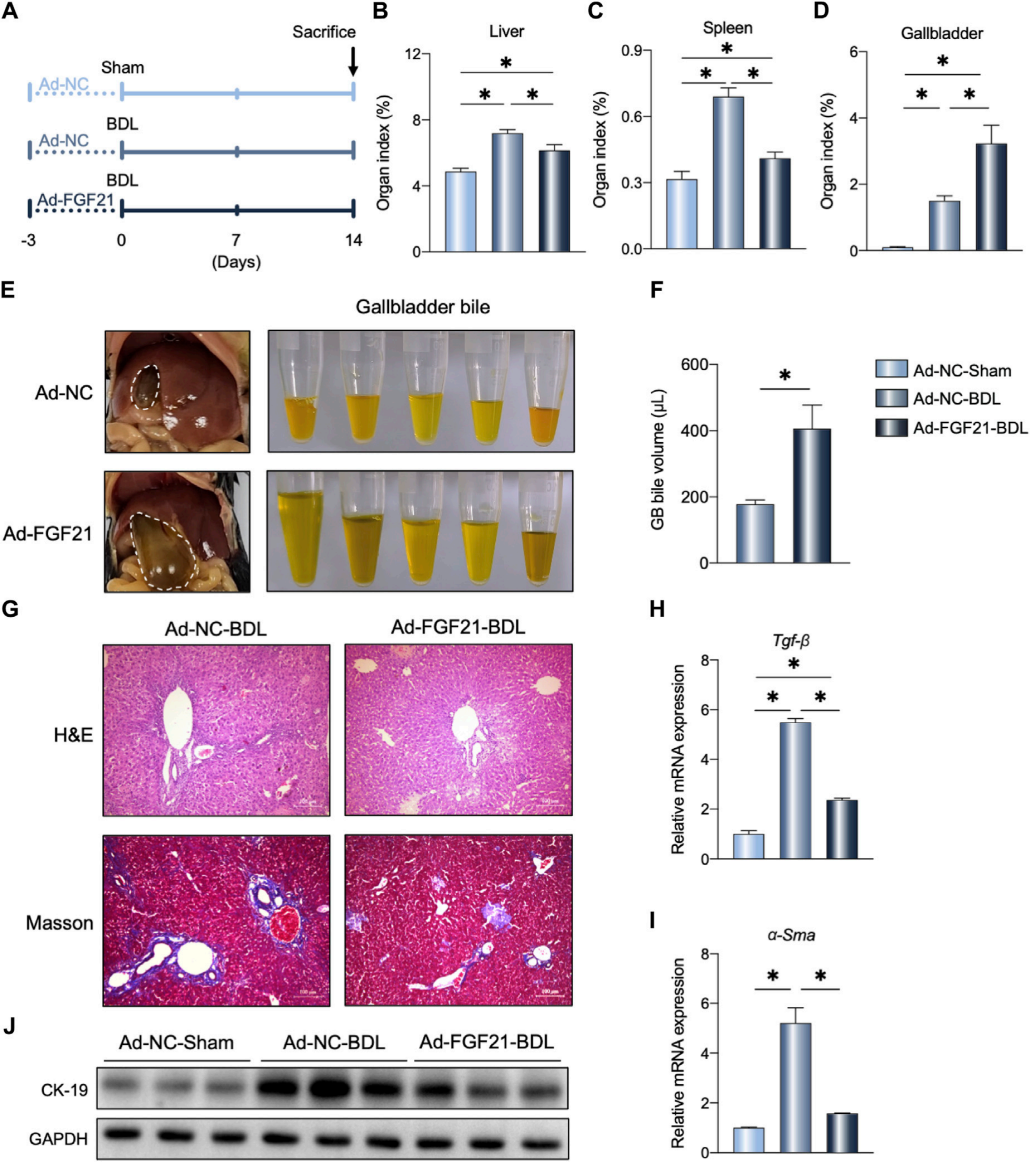
FGF21 overexpression in the WT mice protects against BDL-induced liver fibrosis. **(A)** Experimental outline (n = 5). **(B–D)** Liver, spleen, and gallbladder organ indexes in the indicated groups. **(E, F)** Representative photographs and volume of the gallbladder bile 14 days after BDL. **(G)** Representative images of H&E and Masson staining in the three groups. **(H, I)** Relative mRNA expression levels of *Tgf-β* and *α-Sma* in the liver of the indicated groups. **(J)** Liver protein levels in the three groups. The data were statistically analyzed using ANOVA and Tukey’s multiple comparisons test *p < 0.05.

To confirm the potential therapeutic effects of FGF21 on cholestasis, we administered FGF21 to the WT-BDL mice (Figure 3A; Supplementary Figure S1B, C). As shown in Figure 3B, C, FGF21 overexpression reduced the liver and spleen organ indexes; interestingly, the volume of gallbladder bile also increased in the Ad-FGF21-BDL mice compared to the Ad-NC-BDL mice (Figure 3D–F). Furthermore, histological analysis showed that FGF21 overexpression alleviated BDL-induced liver injury and fibrosis (Figure 3G). The mRNA levels of *Tgf-β* and *α-Sma* also decreased in the Ad-FGF21-BDL mice than the Ad-NC-BDL mice (Figure 3H, I), and the protein levels of CK19 also decreased after FGF21 overexpression (Figure 3J).

### NaB supplementation regulated liver FGF21 expression and gut microbiota composition

**FIGURE 4:**
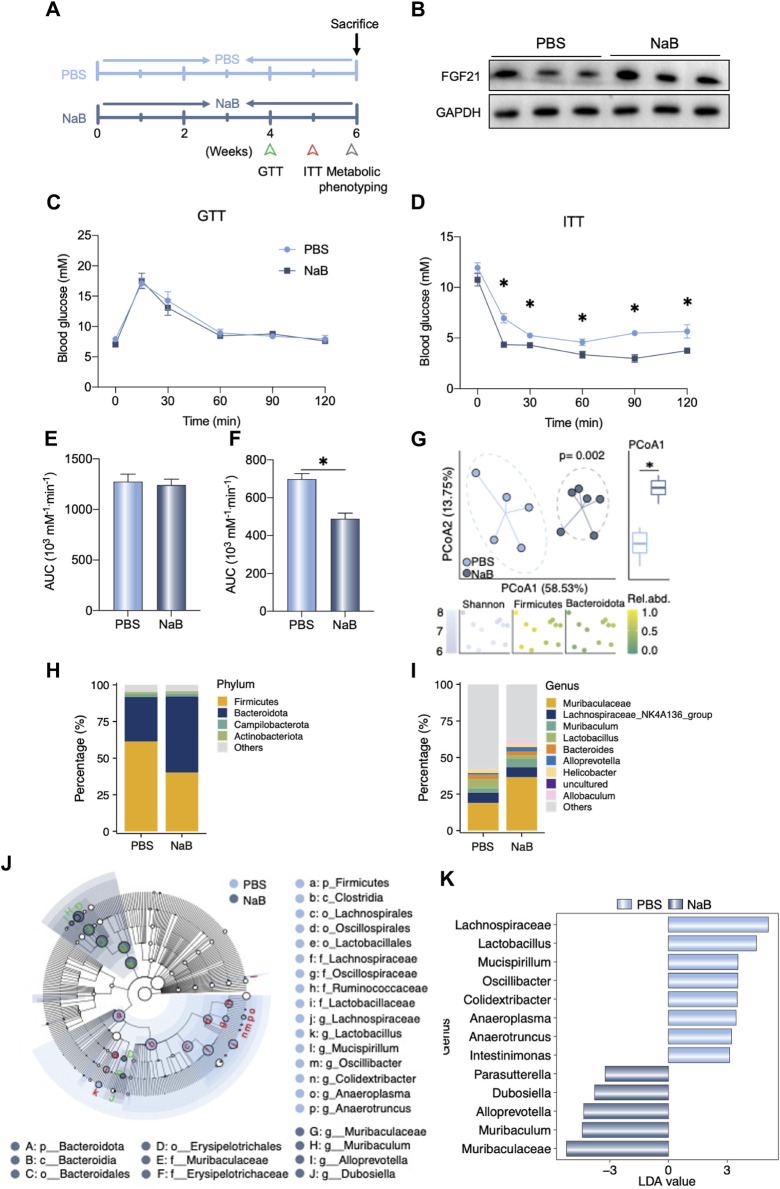
NaB supplementation regulates liver FGF21 expression and gut microbiota composition. **(A)** Experimental outline (n = 5–6). **(B)** Liver FGF21 protein levels in mice after receiving phosphate-buffered saline (PBS) or NaB for 6 weeks. **(C, D)** Glucose tolerance test (GTT) and insulin tolerance test (ITT) were performed in the two groups of mice. **(E, F)** Area under the curve (AUC) of the GTT and ITT data are quantified. **(G)** Beta diversity of the gut microbiota visualized by PCoA based on the Bray–Curtis distance matrix at the genera level. The plot color of the bottom graphic indicates the Shannon index, and the relative abundances of phylum *Firmicutes* and *Bacteroidetes*. In the PCoA1 of Principal Co-ordinates Analysis (PCoA), there was a significant difference between the two groups. **(H, I)** Relative abundances of the gut bacteria at the phylum and genera levels, respectively. **(J)** Cladograms generated on the basis of Linear discriminant analysis Effect Size (LEfSe) analysis indicating the differences in the fecal bacterial taxa. **(K)** Linear discriminant analysis (LDA) values of the LEfSe analysis between the two groups. Only LDA >3 genera are shown. The data were statistically analyzed using unpaired t-test*p < 0.05.

To assess the beneficial effects of NaB, the WT mice were administered NaB for 6 weeks (Figure 4A). As shown in Figure 4B, the hepatic FGF21 protein levels increased after NaB administration; although the ability for glucose metabolism did not change, insulin sensitivity improved significantly after NaB administration (Figures 4C–F). We also analyzed the alterations to the gut microbiota after NaB supplementation. As shown in Figure 4H, the phylum *Firmicutes* decreased after NaB administration but *Bacteroidota* were increased. At the genus level, *Muribaculaceae* and *Muribaculum* were evaluated in the groups with and without NaB supplementation and found to be significantly distinguishable upon Linear discriminant analysis Effect Size (LEfSe) analysis (Figure 4I, J, K); however, *Lachnospiraceae* and *Lactobacillus* decreased after NaB administration (Figure 4I).

### NaB enhanced basic metabolic capacity

**FIGURE 5:**
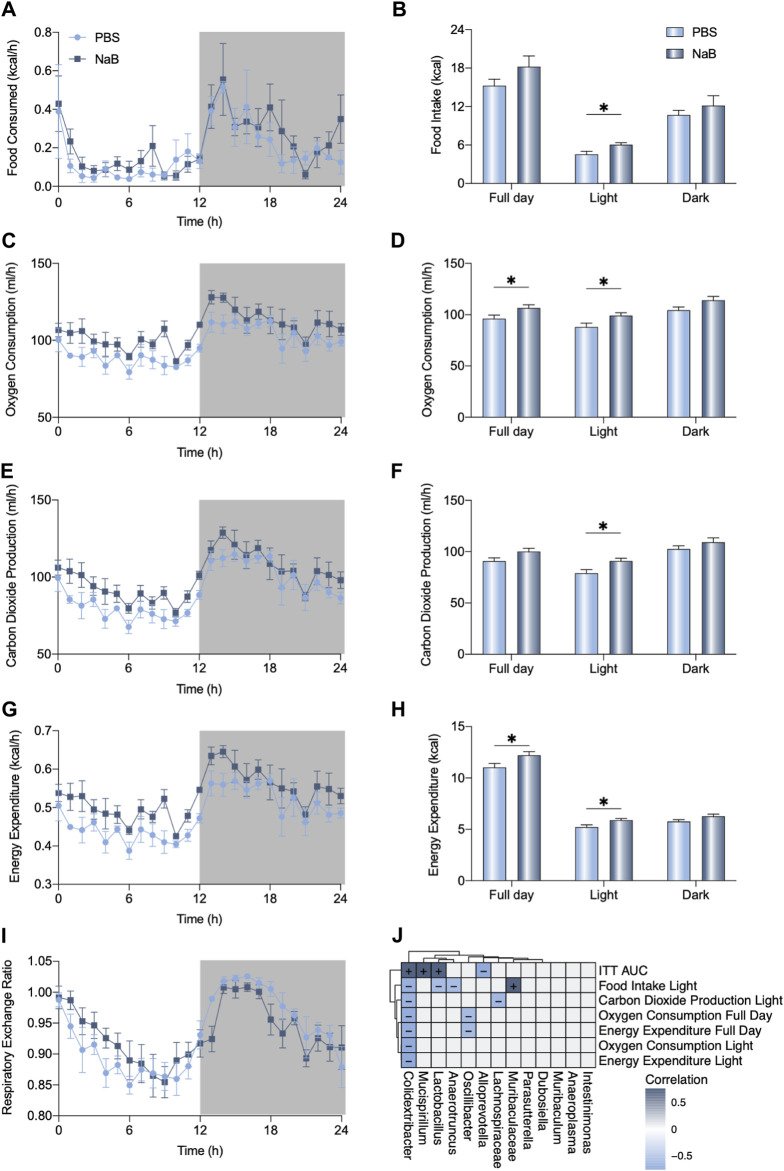
NaB promotes carbohydrate oxidation. **(A, B)** Food consumption, **(C, D)** oxygen consumption, **(E, F)** carbon dioxide production, **(G, H)** energy expenditure, and **(I)** respiratory exchange ratio of each group of mice (n = 5–6) monitored using fully automated metabolic cages during a 24-h cycle (07:00–07:00). **(J)** Correlations between the altered metabolic index with differences in genera (+: positive, -: negative). The data were statistically analyzed using unpaired t-test *p < 0.05.

To determine the metabolic effects of NaB on mice, fully automated metabolic cages were prepared to monitor the metabolic capacities of the mice. As shown in Figure 5A, B, NaB administration significantly increased food intake during the light cycle. However, oxygen consumption and carbon dioxide production rates of the mice increased after NaB administration (Figure 5C–F). The full-day energy expenditure was also evaluated in the mice in the NaB group (Figure 5G, H). The light-cycle respiratory exchange ratios of the NaB group mice were higher than those of the mice in the phosphate-buffered saline (PBS) group but decreased during the night cycle (Figure 5I). The elevated metabolic effects of NaB in the basic condition imply that NaB could promote host resistance after BDL. The enrichment of the *Alloprevotella* genus was negatively correlated with the area under the curve (AUC) of the ITT after NaB administration (Figure 5J); however, *Muribaculaceae* were positively correlated with food intake during the light cycle (Figure 5J). Moreover, enrichment of *Oscillibacter* and *Lachnospiraceae* genus in the PBS group were negatively associated with full-day oxygen consumption and carbon dioxide production during the light cycle, respectively (Figure 5J).

### NaB alleviated BDL-induced liver fibrosis

**FIGURE 6:**
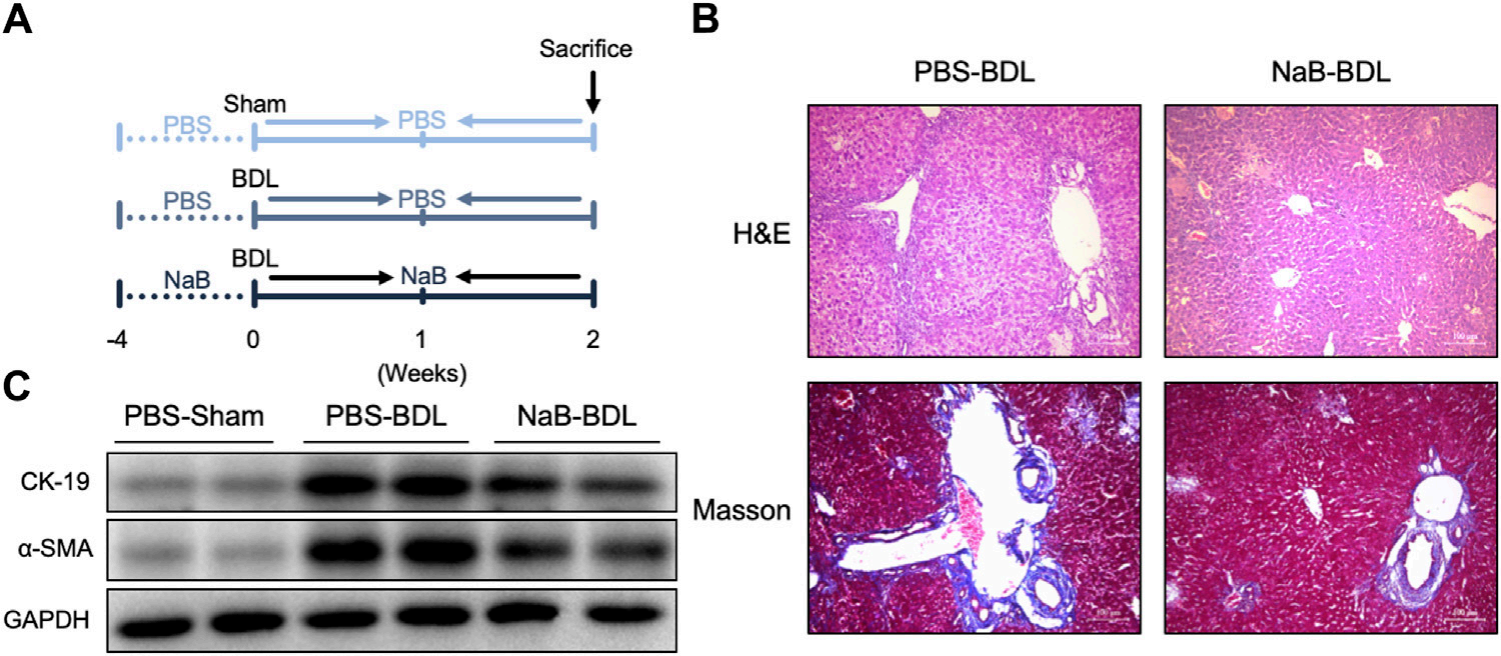
NaB alleviates BDL-induced liver fibrosis in WT mice. **(A)** Experimental outline (n = 5–6). **(B)** Representative images of H&E and Masson staining in the three groups. **(C)** Liver protein levels in the indicated groups.

We primed the mice with NaB for 4 weeks before BDL surgery (Figure 6A). As shown in Figure 6B, these NaB-primed mice showed decreased liver portal area injury and fibrosis after BDL surgery based on histological staining (Figure 6B). Further, the hepatic protein levels of CK19 and α-SMA also decreased in the NaB-primed mice after BDL surgery (Figure 6C).

### NaB administration regulated gut microbiota in an FGF21-dependent manner

**FIGURE 7:**
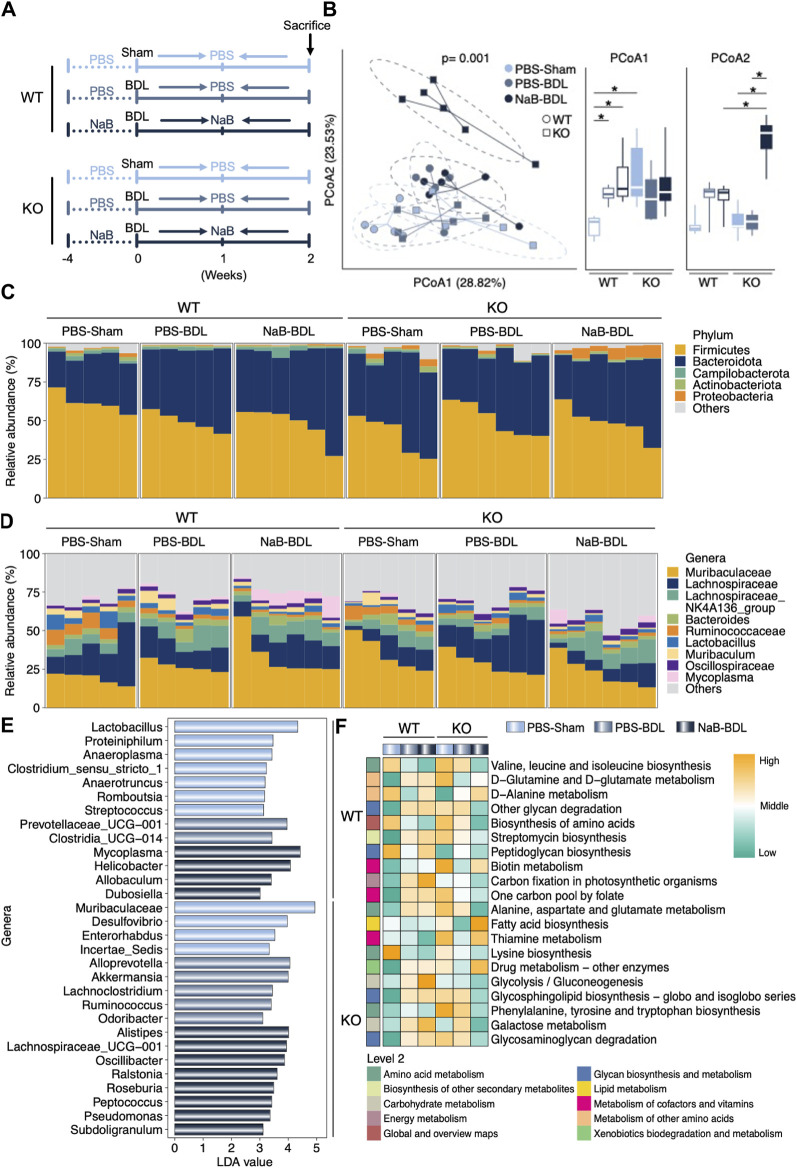
NaB regulates clear differences in the gut microbiota compositions in the WT and FGF21 KO mice that received either sham operation or BDL. **(A)** Experimental outline (n = 5–6). **(B)** Beta diversity of the gut microbiota visualized by PCoA based on the Bray–Curtis distance matrix at the genera level. Both PCoA1 and PCoA2 of PCoA show significant differences among the groups. **(C, D)** Relative abundances of the gut bacteria at the phylum and genera levels, respectively. **(E)** LDA values of the LEfSe analysis for the six groups. Only LDA >3 genera are shown. **(F)** Predicted functions in the indicated groups based on the gut microbiota. The data were statistically analyzed using unpaired t-test*p < 0.05.

We analyzed the gut microbiota alterations in the WT and FGF21 KO mice that received either sham or BDL operations after NaB administration (Figure 7A). As shown in Figure 7B, there were significant differences among the six groups with regard to gut microbiota beta diversity. Specifically, the *Proteobacteria* phylum was enriched in the FGF21 KO mice that received BDL surgery and NaB administration (Figure 7C). At the genus level, NaB supplementation uniquely increased the *Mycoplasma* in the WT-BDL mice rather than KO-BDL mice (Figure 7D). However, NaB administration elevated the abundances of genus *Alistipes*, *Lachnospiraceae_UGG-001*, *Oscillibacter*, and *Ralstonia* in the KO-BDL group (Figure 7E). Peptidoglycan biosynthesis and glycolysis/gluconeogenesis capabilities were also uniquely elevated after NaB administration in the WT-BDL mice rather than the KO-BDL mice (Figure 7F). The predicted gut microbiota functions of fatty acid biosynthesis and thiamine metabolism in the KO-NaB-BDL group were higher those of the WT-NaB-BDL and KO-PBS-BDL groups (Figure 7F).

### NaB alleviated BDL-induced liver fibrosis dependent on FGF21

**FIGURE 8:**
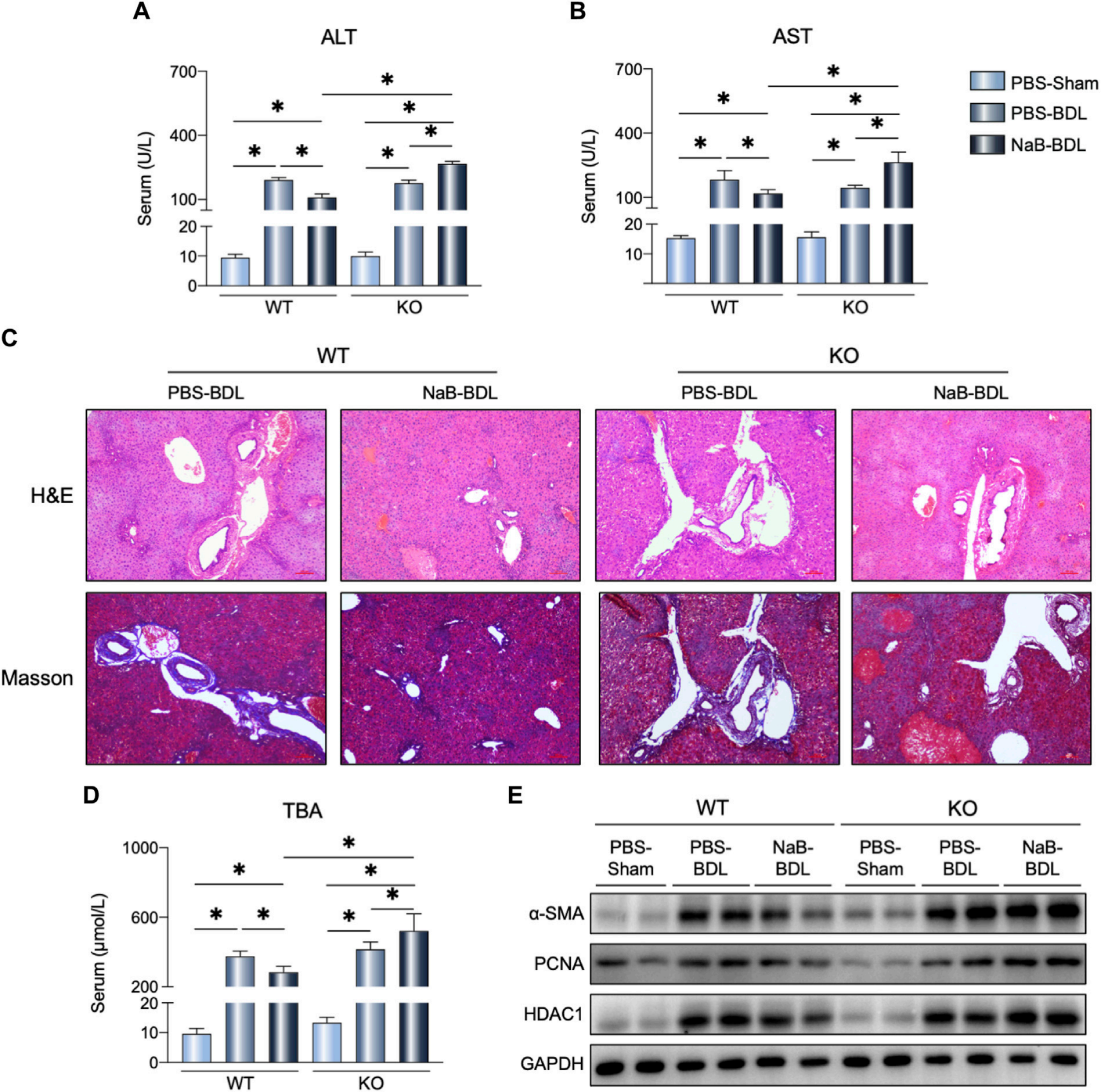
NaB alleviates BDL-induced liver fibrosis by FGF21 expression. **(A, B)** Serum ALT and AST levels in the six groups (n = 5–6). **(C)** Representative images of H&E and Masson staining in the indicated groups. **(D)** Serum TBAs in the six groups. **(E)** Liver protein levels in the indicated groups. The data were statistically analyzed using ANOVA and Tukey’s multiple comparisons test *p < 0.05.

Higher serum ALT and AST levels were observed in the KO-NaB-BDL mice (Figure 8A, B); NaB administration alleviated BDL-induced liver injury and fibrosis in the WT mice rather than the FGF21 KO mice (Figure 8C). In the KO mice, NaB administration increased the serum TBAs concentration compared to the WT-NaB-BDL and KO-PBS-NaB groups (Figure 8D). The protein levels of hepatic α-SMA, PCAN, and HDCA1 in the KO-NaB-BDL group mice were consistently higher than those in the WT-NaB-BDL and KO-PBS-NaB groups (Figure 8E).

## Discussion

The physiological and pathological roles of FGF21 have been reported for multiple chronic and acute liver diseases (Guo et al., 2016; Choi et al., 2023). As a stress-induced hormone, the potential of FGF21 to act as a therapeutic target in the treatment of liver failure has been studied, but the beneficial effects of FGF21 on cholestasis is barely known. We found that liver FGF21 expression level was significantly higher in BDL-induced obstructive cholestasis in mice. This highly induced FGF21 could be a response to the overflow of BAs in the liver, thus preventing excess stress and protecting against liver injury (Cyphert et al., 2012). Moreover, mice with FGF21 deficiency showed more aggravated liver fibrosis and bile duct proliferation in BDL-induced cholestasis for the first time. NaB administration could highly induce hepatic FGF21 to protect against BDL-induced cholestasis, and this beneficial effect of NaB was FGF21-dependent. Together, our results indicate that FGF21 could act as a therapeutic target and that NaB could be an alternative approach for boosting FGF21 signaling in the prevention and treatment of cholestasis.

A previous study reported that FGF21 could ameliorate hepatic fibrosis through multiple mechanisms (Meng et al., 2021). In the obstructive cholestasis model, the excess accumulation of BAs is the principal cause of fibrosis; here, FGF21 could act as a negative regulator of BA synthesis in suppressing the plasma TBAs (Chen et al., 2018). Interestingly, our results revealed that FGF21 overexpression not only reduced liver fibrosis and bile duct proliferation but also expanded the gallbladder volume. However, limited studies are available on the connection between FGF21 and gallbladder function. These findings suggested that FGF21 may decrease liver cholestasis by increasing the expansion ability of the gallbladder, resulting in further alleviation of fibrosis induced by excess BA accumulation.

Owing to the protective effects of FGF21 on cholestasis-induced liver fibrosis, we further analyzed the beneficial effects of NaB, a well-known gut-microbiota-derived SCFA, to induce FGF21 expression in the liver through activation of PPARα (Kundu et al., 2019). As expected, NaB administration not only highly induced the expression of liver FGF21 but also enhanced insulin sensitivity. These data are consistent with the findings of previous studies that FGF21 could modulate glucose homeostasis (Jimenez et al., 2018; Szczepanska and Gietka-Czernel, 2022). Moreover, NaB could increase the *Bacteroidota* phylum in the gut microbiota, which has been identified to enhance the host energy metabolism through metabolization of the fibers in the food, consequently increasing the circulating SCFAs (Trompette et al., 2014). NaB is also known to facilitate the *Alloprevotella* genera, which are positively correlated with insulin sensitivity; a previous study showed that *Alloprevotella* could balance blood glucose levels (Ge et al., 2023). Moreover, NaB administration promotes energy expenditure and the respiratory exchange ratio; accordingly, a previous study showed that NaB supplementation increases the oxidation of fatty acids and carbohydrates (Li et al., 2018). Importantly, NaB inhibits *Colidextribacter,* which is negatively associated with energy expenditure. These findings suggest that NaB pretreatment enhances the energy metabolism of the mice by boosting FGF21 and regulating the gut microbiota.

As noted above, NaB could enhance the host metabolic ability and restore the intestinal microbiota. Therefore, it was not surprising that NaB administration alleviated BDL-induced liver damage. Considering the strong FGF21 induction capability of NaB, we speculated that the beneficial effects of NaB were dependent on the FGF21 signal. As expected, NaB administration in the FGF21-deficient mice receiving BDL operation significantly induced *Proteobacteria* phylum proportion as it particularly accounts for many pathogens (Jiang et al., 2017). More importantly, NaB treatment aggravated BDL-indued liver injury and fibrosis in the FGF21 KO mice. Numerous studies have demonstrated that NaB has beneficial effects for multiple metabolic diseases (Sun et al., 2018). However, in some cases, the overproduction of SCFAs can become unfavorable to the host (Serino, 2019); for example, increased propionate levels in the feces are related to increased risk of type 2 diabetes (Sanna et al., 2019). Importantly, our results indicate that NaB has detrimental effects on cholestasis mice with FGF21 deficiency. Thus, we speculate that higher levels of circuiting NaB could induce liver FGF21 expression, which in return could enhance the capability of NaB oxidation. However, in the FGF21-deficient mice, NaB administration did not facilitate NaB clearance via FGF21, consequently resulting in a high plasma concentration. As described previously, the higher plasma SCFA concentrations could be potentially harmful (van der Beek et al., 2015); therefore, the host FGF21 genetic expression should be taken into consideration before NaB application for disease treatment. More importantly, the short half-life of FGF21 poses challenges to its translational potential in clinical settings (Hu et al., 2020). Our results thus indicate that NaB administration could be an alternative approach to boosting FGF21 signaling and regulating gut microbiota in the treatment of cholestasis.

Although the above findings are encouraging, there are some limitations to our study. First, FGF21 overexpression alleviates BDL-induced liver fibrosis and bile duct proliferation accompanied by gallbladder volume expansion. To explore whether FGF21 alleviated BDL-induced liver damage depending on the gallbladder, a cholecystectomy should be considered in future research. Second, given that cholestasis is often caused by BA transportation and resorption dysbiosis, the BA pool and related gene expressions in treatment with FGF21 or NaB need to be studied further. Lastly, fecal microbiota transplantation from NaB-supplemented mice to BDL mice may be a possible strategy to confirm the beneficial effects of NaB on cholestasis through modulation of gut microbiota, even as this study shows that NaB administration could alleviate cholestasis via increasing FGF21 expression and regulating gut microbiota.

In conclusion, the present study shows that FGF21 could protect the host against BDL-induced liver injury and fibrosis and that NaB administration can enhance host metabolic capability by inducing liver FGF21 expression and restoring the gut microbiota. Importantly, this study identifies that NaB could alleviate BDL-induced liver damage and fibrosis in an FGF21-dependent manner. These comprehensive findings suggest that NaB administration can be a novel nutritional therapy for treating cholestasis by boosting FGF21 signaling and regulating the gut microbiota.

## Data Availability

The data presented in the study are deposited in the NCBI repository, accession number PRJNA1127873.

## Abbreviations

ALT, serum alanine aminotransferase; AST, aspartate transaminase; BDL, bile duct ligation; BA, bile acid; FGF21, fibroblast growth factor 21; FXR, farnesoid X receptor; MCD, methionine and choline deficient L-amino acid diet; NaB, sodium butyrate; OCA, obeticholic acid; SCFA, short-chain fatty acid.
