# Supplementary figures and images for: FGF21-dependent alleviation of cholestasis-induced liver fibrosis by sodium butyrate
Source: Front Pharmacol. 2024 Jul 8;15:1422770. doi: 10.3389/fphar.2024.1422770 (PMC11260614; doi:10.3389/fphar.2024.1422770)

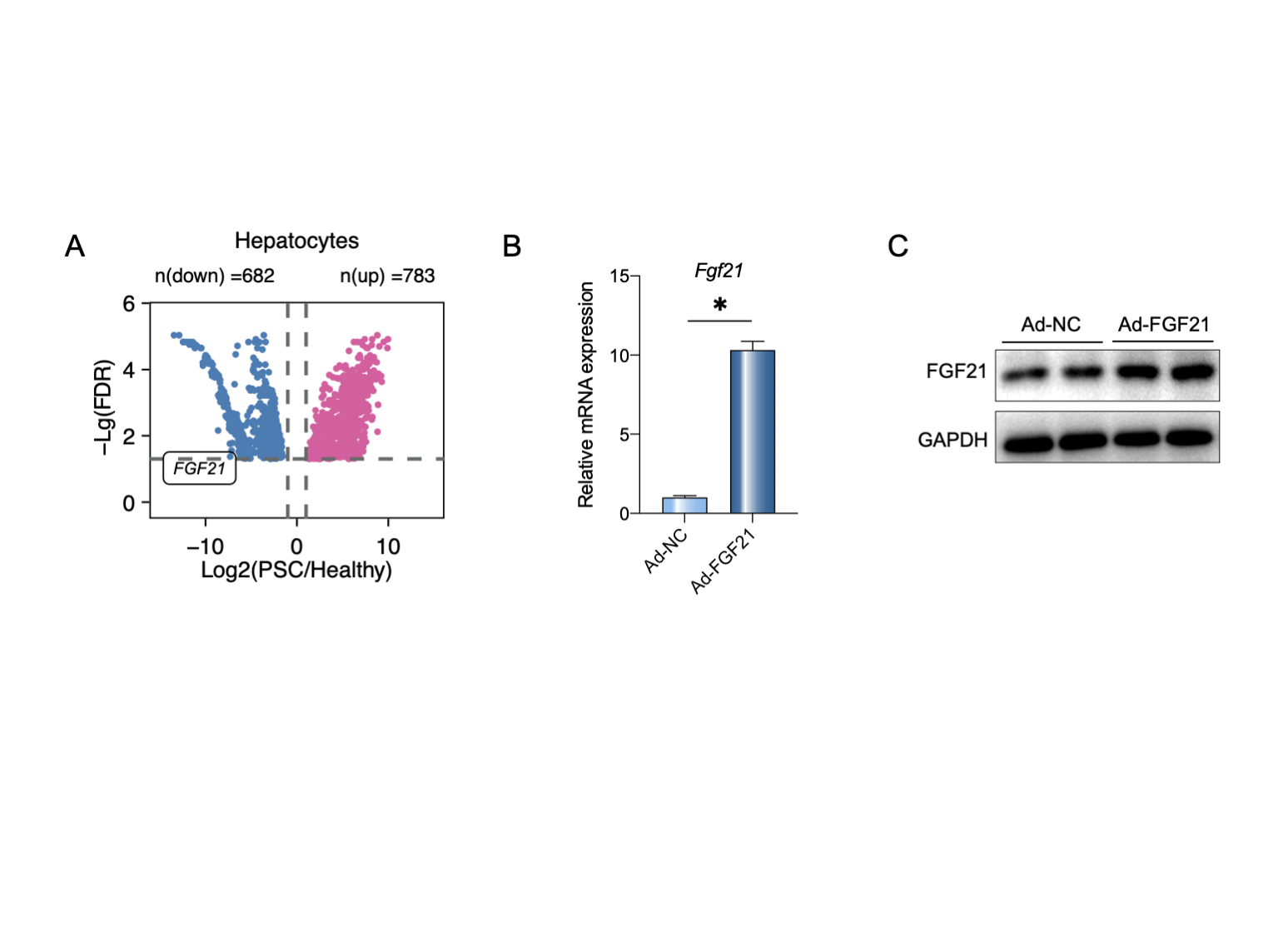

Supplement: Supplementary file 1 [file Image1.TIFF]
